# P-2082. Insurance-Related Interruptions and Risk of Discontinuation of Long-Acting Injectable CAB/RPV in a South Side Chicago HIV Clinic

**DOI:** 10.1093/ofid/ofaf695.2246

**Published:** 2026-01-11

**Authors:** Lauren S Kang, Christopher Kaperak, Eleanor E Friedman, Moira McNulty, Aniruddha Hazra

**Affiliations:** The University of Chicago Pritzker School of Medicine, Chicago, IL; University of Chicago Medicine, Chicago, IL; University of Chicago, Chicago, Illinois; University of Chicago, Chicago, Illinois; University of Chicago, Chicago, Illinois

## Abstract

**Background:**

Adherence to daily oral antiretroviral therapy among people with HIV (PWH) is often hindered by structural and psychosocial barriers. Long-acting injectable cabotegravir/rilpivirine (LAI-CAB/RPV) offers a promising alternative, although systemic challenges—particularly insurance instability—may limit sustained access. We evaluated real-world outcomes, specifically delayed or missed injections and permanent discontinuations, among patients referred for LAI-CAB/RPV at a Ryan White-funded HIV clinic on Chicago’s South Side.

**Methods:**

We included PWH aged ≥18 years with ≥1 HIV clinic visit and ≥2 viral load measurements between January 1, 2021, and January 31, 2025. Manual chart review captured injection dates and reasons for interruptions and discontinuations. Comparative analyses were conducted before and after May 31, 2023, when the initial study period was expanded. Categorical associations with treatment interruptions and discontinuations were assessed using Fisher’s exact test.

**Results:**

A total of 127 individuals initiated LAI-CAB/RPV during the study period. Patients who started after May 31, 2023 were significantly less likely to experience treatment interruptions (93.5% vs. 61.7% without interruptions; OR 0.11, *p*< 0.001) or discontinuations (2.2% vs. 24.7%; OR 14.75, *p*< 0.001). Insurance-related barriers were the most common reason for both outcomes. Experiencing issues with coverage or access was significantly associated with interruptions (*p*< 0.001); 46% of those with an interruption had documented insurance issues. Nearly one in five discontinuations (19.0%) occurred in the context of insurance barriers, though this association was not statistically significant. Notably, treatment interruption was significantly associated with subsequent discontinuation (OR 3.43, *p*=0.02).

**Conclusion:**

These findings highlight the critical impact of insurance-related barriers on the continuity of LAI-CAB/RPV. Despite improvements over time, systemic access challenges remain a major contributor to treatment interruption and are strongly associated with LAI-CAB/RPV discontinuation. Targeted structural interventions are urgently needed to ensure equitable and sustained implementation of long-acting HIV therapies.

**Disclosures:**

Aniruddha Hazra, MD, Gilead Sciences: Advisor/Consultant|Gilead Sciences: Grant/Research Support|ViiV Healthcare: Advisor/Consultant

